# Characterizing entanglement of an artificial atom and a cavity cat state with Bell's inequality

**DOI:** 10.1038/ncomms9970

**Published:** 2015-11-27

**Authors:** Brian Vlastakis, Andrei Petrenko, Nissim Ofek, Luyan Sun, Zaki Leghtas, Katrina Sliwa, Yehan Liu, Michael Hatridge, Jacob Blumoff, Luigi Frunzio, Mazyar Mirrahimi, Liang Jiang, M. H. Devoret, R. J. Schoelkopf

**Affiliations:** 1 Departments of Physics and Applied Physics, Yale University, New Haven, Connecticut 06510, USA; 2 INRIA Paris-Rocquencourt, Domaine de Voluceau, B.P. 105, 78153 Le Chesnay Cedex, France

## Abstract

The Schrodinger's cat thought experiment highlights the counterintuitive concept of entanglement in macroscopically distinguishable systems. The hallmark of entanglement is the detection of strong correlations between systems, most starkly demonstrated by the violation of a Bell inequality. No violation of a Bell inequality has been observed for a system entangled with a superposition of coherent states, known as a cat state. Here we use the Clauser–Horne–Shimony–Holt formulation of a Bell test to characterize entanglement between an artificial atom and a cat state, or a Bell-cat. Using superconducting circuits with high-fidelity measurements and real-time feedback, we detect correlations that surpass the classical maximum of the Bell inequality. We investigate the influence of decoherence with states up to 16 photons in size and characterize the system by introducing joint Wigner tomography. Such techniques demonstrate that information stored in superpositions of coherent states can be extracted efficiently, a crucial requirement for quantum computing with resonators.

## Abstract (web summary)

Qubit-cavity entanglement can be used for quantum information processing and for investigating the quantum-to-classical transition with high control. Here, the authors characterize the entanglement between an artificial atom and a cat state and its susceptibility to decoherence through Bell test witnesses.

## Graphical abstract

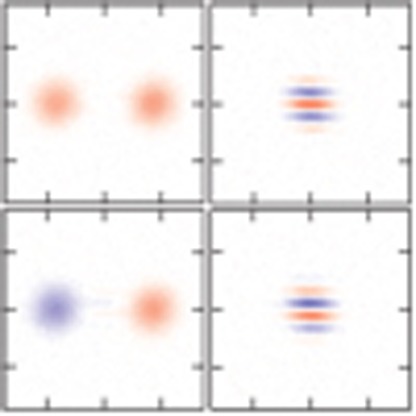

Quantum information processing necessitates the creation and detection of complex entangled states. Many physical implementations aim to encode quantum information into large registers of entangled two-level systems, or qubits. Although originally proposed to investigate local hidden variable theory^1^, a Bell inequality can be used to benchmark the ability to entangle and extract information from an entangled two-qubit system^2^. Using the Clauser–Horne–Shimony–Holt (CHSH) variant^3^ of the Bell test, this violation has been demonstrated with photons^4^,^5^, atoms^6^,^7^, solid-state spins^8^ and artificial atoms in superconducting circuits^9^,^10^. However, quantum computation necessitates the entanglement of large numbers of qubits. To perform tasks such as quantum error correction, a physical implementation must be capable of high-fidelity multi-qubit entanglement, as well as the efficient detection multi-qubit observables. For these larger, more distinguishable states, creating and preserving entanglement becomes increasingly difficult due to the rapid onset of decoherence^11^. Alternative encoding schemes that use coherent state superpositions, known as cat states^12^, take advantage of a cavity resonators much larger Hilbert space, as compared with that of a two-level system. This architecture allows redundant qubit encodings that can simplify the operations needed to initialize, manipulate and measure the encoded information^13^,^14^,^15^. For such a system to be viable as a quantum computing platform, efficient measurement of such encoded qubit observables must be possible. Using a circuit quantum electrodynamics architecture^16^, we show efficient, high-fidelity measurements of an encoded cat state qubit and demonstrate this technology by detecting a violation of the CHSH Bell inequality between the encoded cat state qubit and a superconducting transmon qubit^17^. Furthermore, by the use of coherent states in this composite system, we can investigate the effects of decoherence by continuously varying the size of prepared entangled states^18^,^19^, something unachievable with discrete systems. These techniques provide an important set of analytical tools for quantum systems composed of entangled qubits and resonators^14^,^19^,^20^,^21^,^22^,^23^, and demonstrate that one can exploit coherent state superpositions in resonators without sacrificing measurement efficiency.

A resonator state can be completely described by direct measurements in the continuous variable basis with the cavity state Wigner function^24^. We extend this concept to express an entangled qubit–cavity state in what we call the joint Wigner representation. We construct this representation by performing a sequence of two quantum non-demolition measurements (Fig. 1), where a qubit state measurement is correlated with a subsequent cavity state measurement. However, complete cavity state tomography need not be required, and in fact many fewer measurements could be used to characterize a state when operating in a smaller, encoded subspace. By choosing an encoding scheme where states of a quantum bit are mapped onto a superposition of coherent states 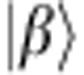 and 
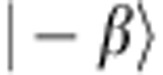
, we can condense the joint Wigner representation down to just 16 correlations, equivalent to a two-qubit measurement set. Using direct fidelity estimation (DFE)^25^,^26^ and CHSH Bell witnesses^27^,^28^ within this logical basis, we investigate this systems susceptibility to decoherence by continuously increasing the cat state amplitude *β*. We measure a range in which correlations surpass the Bell inequality threshold and observe its reduction due to decoherence, benchmarking the efficiency of our encoding and detection schemes with cat state qubits.

## Results

### Creating the Bell-cat state

**Figure 1:**
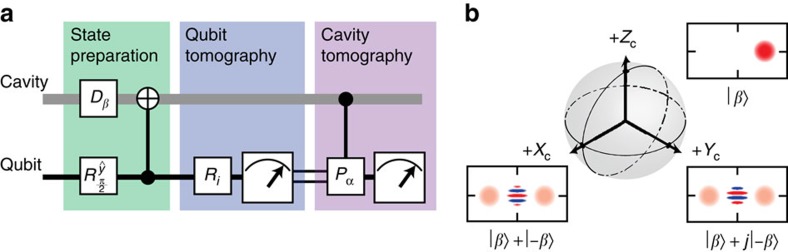
Sequential detection for entanglement characterization. (**a**) A quantum circuit outlines the method to prepare and measure entanglement between a qubit and cavity state using sequential detection. State preparation is performed by first creating a product state 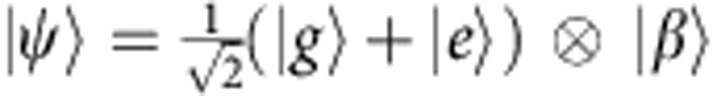 with a cavity displacement *D*_*β*_ of amplitude *β* and a qubit gate 
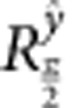
 corresponding to a 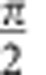 rotation around the 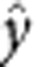 axis. A conditional gate using the dispersive interaction produces the entangled state 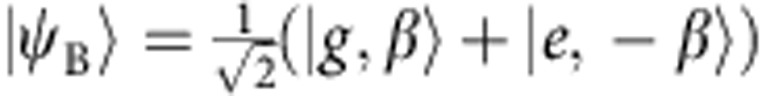. Tomography is performed by measuring an observable of both the qubit and cavity with sequential quantum non-demolition measurements. A pre-rotation *R*_*i*_ allows qubit detection along one of three basis vectors *X*, *Y* and *Z*. The qubit is reset and a cavity observable *P*_*α*_ is mapped to the qubit for a subsequent measurement, where 
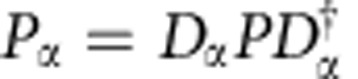
 is the displaced photon number parity operator. Sequential detections are binary results compared shot-by-shot to determine qubit–cavity correlations. (**b**) The space spanned by the superposition of quasi-orthogonal coherent states 
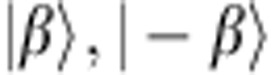
 constitutes an encoded quantum bit in the cavity. While the cavity state can be represented by its Wigner function, this logical state is also described by a vector within its encoded Bloch sphere. Shown is the encoded qubit bloch sphere denoting the +*X*_c_, +*Y*_c_ and +*Z*_c_ encoded states; a diagram of the cavity Wigner function accompanies each of these three states. For well-separated coherent state superpositions, the entangled state 
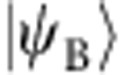
 is then equivalent to a two-qubit Bell state.

This experiment utilizes a circuit quantum electrodynamics architecture^16^,^17^ consisting of two waveguide cavities coupled to a single transmon qubit^22^,^29^. One long-lived cavity (relaxation time *τ*_s_=55 μms) is used for quantum information storage, while the other cavity, with fast field decay (relaxation time *τ*_r_=30 ns), is used to realize repeated measurements. A transmon qubit (relaxation and decoherence times *T*_1_, *T*_2_≈10 μs) is coupled to both cavity modes and mediates entanglement and measurement of the storage cavity state. All modes have transition frequencies between 5–8 GHz and are off-resonantly coupled. The storage cavity and qubit mode are well described by the dispersive Hamiltonian:


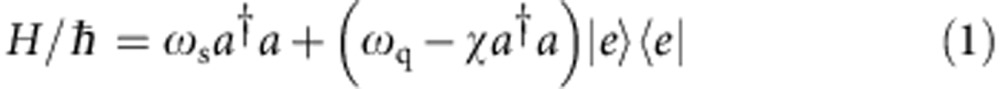


where *a* is the storage cavity ladder operator, 
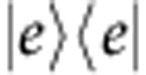
 is the excited state qubit projector, *ω*_s_ and *ω*_q_ are the storage cavity and qubit transition frequencies, and *χ* is the dispersive interaction strength between the two modes (1.4 MHz). This interaction creates a shift in the transition frequency of one mode dependent on the other's excitation number, resulting in qubit–cavity entanglement^30^. As described in Fig. 1, the system is first prepared in a product state 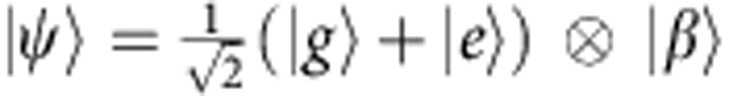, where 
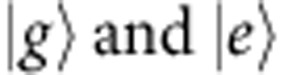
 are the ground and excited states of the qubit, and 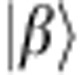 is a coherent state of the cavity mode. Under the dispersive interaction, we allow the system to evolve for a time 
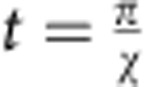
, creating the entangled state:


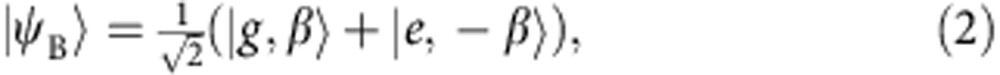


which we call a Bell-cat state^22^,^29^,^30^, mirroring the form of a two-qubit Bell state (for example, 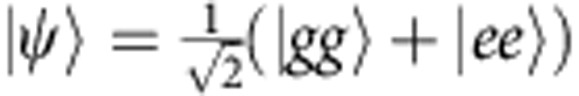).

Correlating sequential high-fidelity measurements of the qubit and cavity allows state tomography of this composite system. We use a Josephson bifurcation amplifier^31^ in a double-pumped configuration in combination^32^,^33^ with a dispersive readout to perform repeated quantum non-demolition measurements with qubit detection fidelity of 98.0% at a minimum of 800 ns between measurements. With this sequence of two measurements, we characterize the efficacy of our entangling scheme and efficiency of measuring qubit–cavity observables with joint Wigner tomography, DFE and a CHSH inequality. The results of these tests illustrate our ability to recast the state encoded in the cavity as one that has a small, simple set of observables that directly mirrors that of the physical qubit.

### Joint Wigner tomography

**Figure 2:**
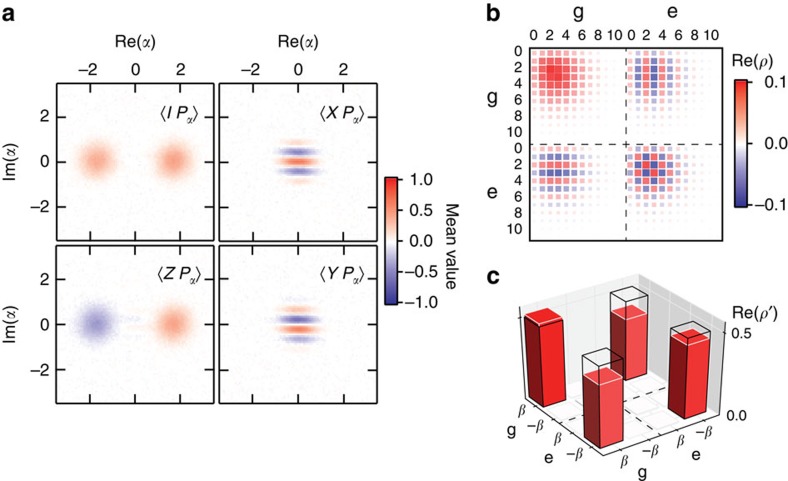
Joint Wigner tomography of a Bell-cat state. (**a**) The set of joint Wigner functions 
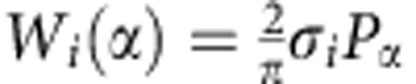
 represents the state of a qubit–cavity system with correlations between the qubit observables *σ*_*i*_={*I*, *X*, *Y*, *Z*} and cavity observable *P*_*α*_ reported for a state 
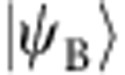
 and displacement amplitude 
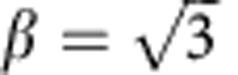
. Shown are measurements comprised of four panels *IP*_*α*_, *XP*_*α*_, *YP*_*α*_ and *ZP*_*α*_ of 6,500 correlations each between the qubit and cavity states. Interference fringes in *XP*_*α*_ and *YP*_*α*_ reveal quantum coherence in the entangled state. (**b**) From the set of joint Wigner functions, we performed a density matrix reconstruction to show the combined qubit–cavity state *ρ* in the Fock state basis. (**c**) Projecting *ρ* onto the logical basis 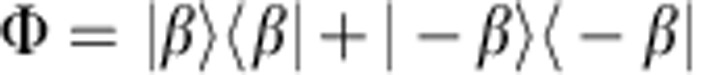, produces the reduced, unnormalized density matrix *ρ*′=Φ*ρ*Φ^†^ in the form of a traditional Bell state. The reduction in contrast of the off-diagonal components in *ρ*′ is due to decoherence in the physical system during preparation and measurement.

The first measurement detects the qubit along one of its basis vectors {*X*, *Y*, *Z*}. This value is recorded and the qubit is reset to 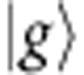 using real-time feedback. The displaced photon number parity observable *P*_*α*_ of the cavity is subsequently mapped onto the qubit using Ramsey interferometry^24^ before a second qubit state detection. The cavity observable 
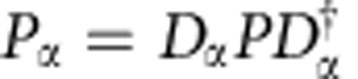
, where *D*_*α*_ is the displacement operator and *P* the photon number parity operator, is detected with 95.5% fidelity. The Wigner function 
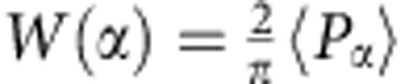
 is constructed from an ensemble of such measurements with different displacement amplitudes *α*. The correlations between the qubit and cavity states make up what we refer to as the joint Wigner functions:


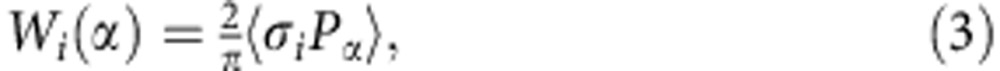


where *σ*_*i*_ is an observable in the qubit Pauli set {*I*, *X*, *Y*, *Z*}. These four distributions are a complete representation of the combined qubit–cavity quantum state (Fig. 2). While other representations exist for similar systems^34^,^35^,^36^,^37^, *W*_*i*_(*α*) is directly measured with this detection scheme and does not require a density matrix reconstruction. By an overlap integral (Supplementary Note 4), we determine the fidelity to a target state 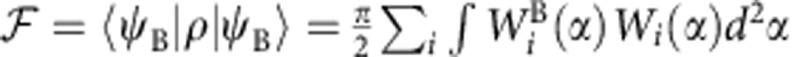, where 
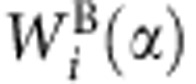
 are the joint Wigner functions of the ideal state, 
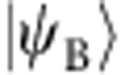
 and *W*_*i*_(*α*) are the measured joint Wigner functions (normalized), yielding a state fidelity 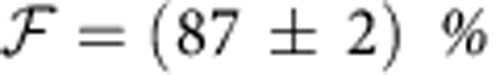 for a displacement amplitude 
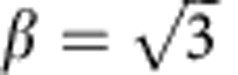
. This amplitude was chosen to ensure orthogonality between logical states 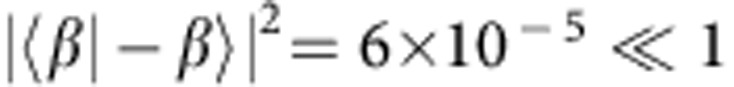 with minimal tradeoff due to photon loss. Furthermore, the efficiency of our detection scheme can be quantified by the visibility of the unnormalized joint Wigner measurements 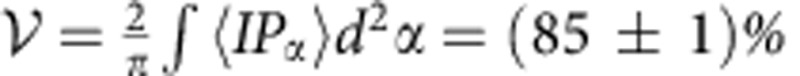. Visibility 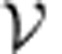 is primarily limited by measurement fidelity and qubit decoherence between detection events (Supplementary Note 5). The parameters 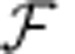 and 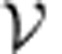 represent critical benchmarks for creating and retrieving information from entangled states.

### Direct fidelity estimation

**Figure 3:**
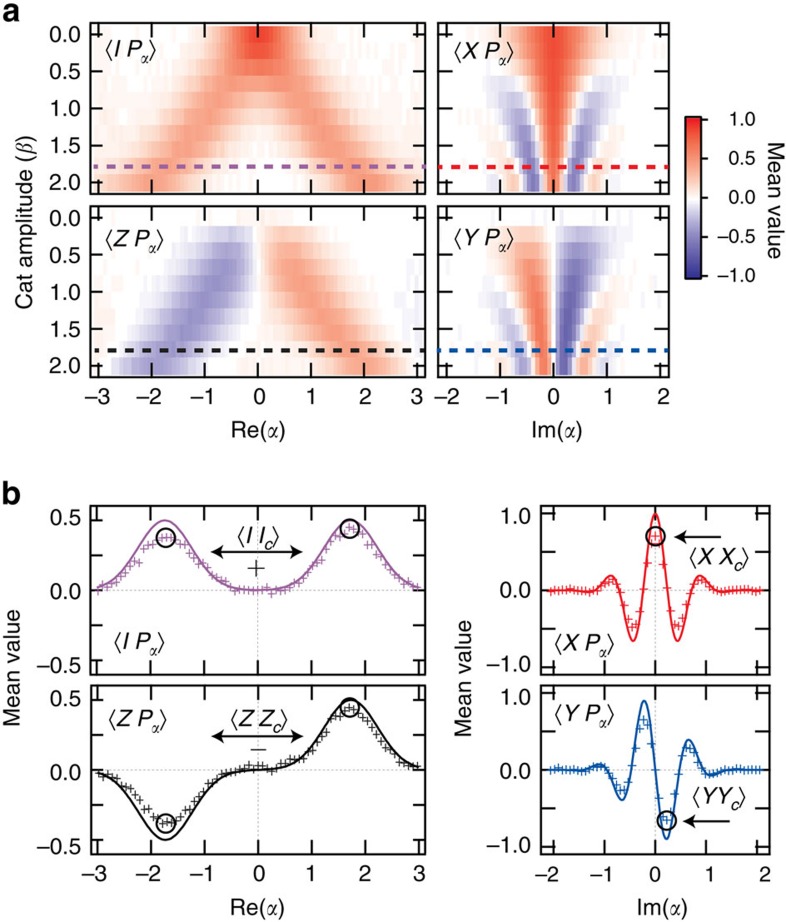
Qubit–cavity correlations. (**a**) Correlations are measured for entangled states 
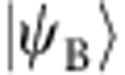
 with cat state amplitudes ranging from *β*=0 to 2. Cuts in joint Wigner functions *IP*_α_ and *ZP*_*α*_ at Im(*α*)=0 show the increasing separation of the coherent state superpositions. Cuts in the joint Wigner functions *XP*_*α*_ and *YP*_*α*_ at Re(*α*)=0 reveal the interference fringe oscillations dependence on cat state size, which increase in frequency with increasing cat state amplitude. (**b**) By viewing just single cuts at 
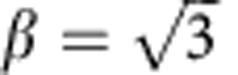
, we see single-shot correlations (crosses) as compared with what is expected from an ideal system with perfect preparation and measurement (solid line). From the cuts in **b** we see the individual measurement settings used to determine joint encoded observables {*II*_c_, *XX*_c_, *YY*_c_, *ZZ*_c_}. While *XX*_*c*_ and *YY*_*c*_ can be determined from a single measurement setting, *II*_c_ and *ZZ*_c_ are determined from the sum and difference of two different settings. From these four correlations, we immediately find a fidelity to an entangled state 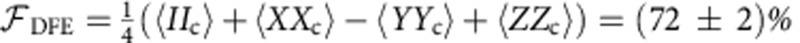.

The number of measurement settings required to perform cavity state tomography can be resource intensive. Restricting to an encoded qubit subspace, only four values of the cavity Wigner function *W*(*α*) are required to reconstruct the state, known as a DFE^25^,^26^. For large cat states 
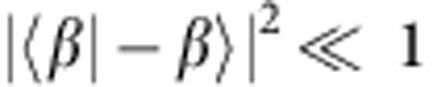
, the encoded state observables map to cavity observables as:


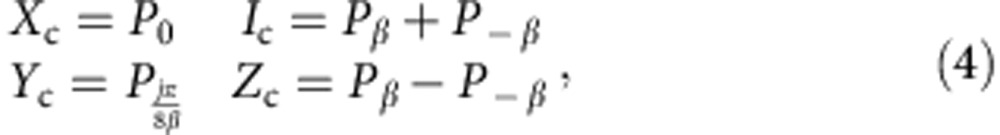


where {*I*_c_, *X*_c_, *Y*_c_, *Z*_c_} form the Pauli set for the encoded qubit state in the cavity (Supplementary Note 8). Cuts in the joint Wigner function (Fig. 3) show these observables and their correlations to the qubit as a function of cat state size. As the superposition state is made larger, interference fringe oscillations increase, while fringe amplitude decreases due to photon loss. For a state 
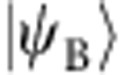
 with 
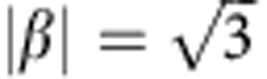
, we estimate a direct fidelity 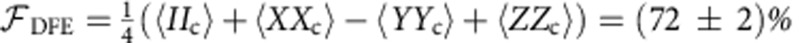 putting a fidelity bound on the target state with no corrections for visibility. This estimate is related to the benchmarks reported above 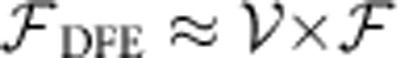 and far surpasses the 50% threshold for a classically correlated state. This indicates both high-fidelity state preparation and measurement, and demonstrates that strong correlations are directly detectable using joint Wigner tomography.

### Bell inequality measurements

**Figure 4:**
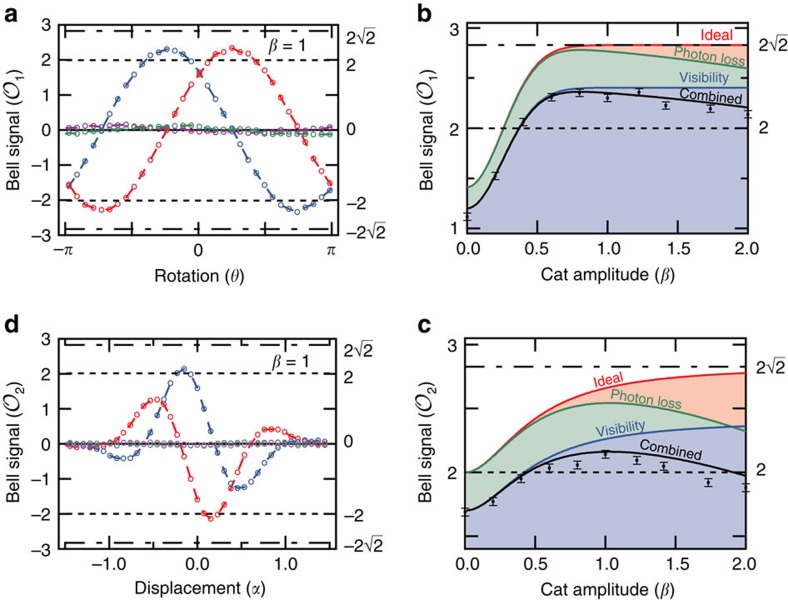
Bell tests with a cat state. A CHSH Bell test between a qubit and cavity is the sum of four correlations 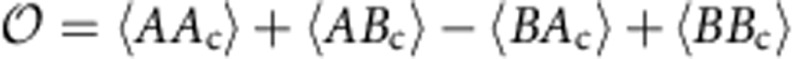, where *A* and *B* are observables of the qubit, and *A*_*c*_ and *B*_*c*_ are observables of the cavity. (**a**) We use correlations between qubit state observables *X*(*θ*)=*X* cos (*θ*/2)+*Z* sin (*θ*/2) and *Z*(*θ*)=*Z* cos (*θ*/2)−*X* sin (*θ*/2) and encoded state observables *X*_c_ and *Z*_c_ to perform a CHSH Bell test as a function of qubit detector angle *θ*. Shown in **a** are four traces that are the result of every possible combination of *X*, *Z*, *X*_c_ and *Z*_c_. A maximum Bell signal is found at 
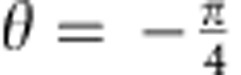
. (**b**) We report this maximum Bell signal for different cat state amplitudes *β*. Plotted points (black) are the average Bell signal for a given amplitude and show the dependence of the entangled state with photon loss and detector visibility. Error bars denote the s.d. of the average signal due to random error as a consequence of a limited sample size (*N*=4,000). Solid lines describe the predicted trends given the measured cavity decay rate and detection visibility. While the ideal behaviour (red) for an entangled state approaches 
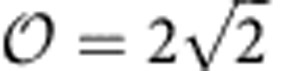
, photon loss (green), detector visibility (blue) and their combined effects (black) will ultimately limit the maximum Bell signal achieved. (**c**,**d**) Furthermore, we realize a second Bell test using qubit observables *X* and *Y*, and cavity state observables 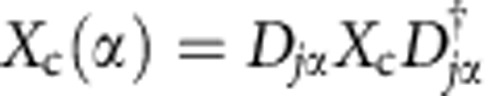 and 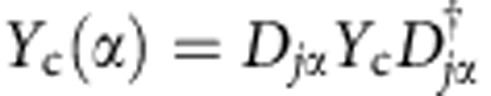, where *α* corresponds to a tomography displacement amplitude serving as a rotation of the effective cavity detector angle. There is a mismatch in the maxima obtained in the two different Bell tests due to increased susceptibility to photon loss in the second test. (**c**,**d**) Both, however, show a violation at least four s.d.'s beyond the classical limit defined by the CHSH Bell inequality.

To place a stricter bound on observed entanglement, we perform a Bell test on the measured state. Although proposed to investigate local hidden variable theory, the Bell test here serves to benchmark the performance of a system that creates and measures entangled states^8^,^9^,^10^. Bell tests using homodyne measurements have been proposed^38^,^39^; however, here we choose the CHSH Bell test which states that the sum of four classical correlations will be bounded such that:


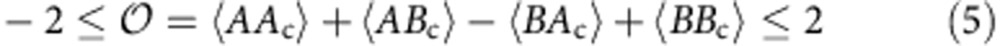


where, in this experiment, *A* and *B* are two qubit observables and *A*_c_ and *B*_c_ are two cavity observables. We perform two Bell tests (Fig. 4) with correlations taken shot by shot with no post selection or compensation for detector inefficiencies. In the first, we take observables *X*(*θ*)=*X*cos(*θ*/2)+*Z*sin(*θ*/2), *Z*(*θ*)=*Z*cos(*θ*/2)−*X*sin(*θ*/2), *X*_c_, *Z*_c_ and sweep both qubit detector angle *θ* (Supplementary Note 11) and cat state amplitude *β*. We observe a Bell signal with a maximal value 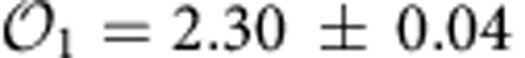 at 
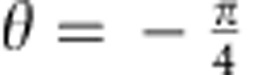
 for *β*=1. We witness a Bell signal surpassing bounded values up to cat states of size 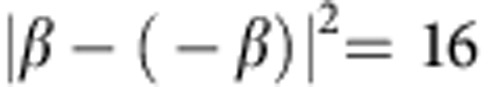 photons^19^,^29^.

Measurements along *Z*_c_ require assumptions on the symmetry of the prepared state (Supplementary Note 8); we can instead employ an alternative Bell test. Using a scheme similar to ref. 28, and choosing observables 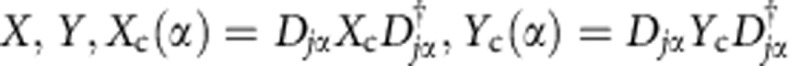, where *α* is a displacement amplitude corresponding to a rotation of the encoded cavity state detector (Supplementary Note 11), we observe a maximal value 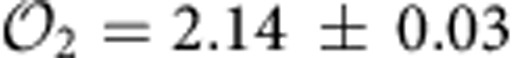 for *β*=1. A lower Bell signal is observed in the second test due to its greater sensitivity to photon loss, yet in both tests two regimes are evident. For small cat state amplitudes, the initial Bell signal is limited by the non-orthogonality of the coherent state superpositions (Supplementary Note 7), while for large displacements the system's sensitivity to photon loss results in a reduction of the Bell signal. Larger, more distinguishable states quickly devolve into a classical mixture due to the onset of decoherence, corresponding to the resolution of Schrödinger's thought experiment. However, for intermediate cat state sizes, we observe Bell signals surpassing classical predictions larger than statistical uncertainties in both tests.

## Discussion

In this letter, we have demonstrated the efficient detection of an artificial atom and a cat state in a cavity mode. We determine the entangled state using sequential detection with high-fidelity state measurement and real-time feedback on the quantum state. We benchmark the capabilities of this detection scheme with DFE and Bell test witnesses, which both reveal non-classical correlations of our system. Besides characterizing the high degree of entanglement in our Bell-cat, the tests detailed above also demonstrate that simple encoding techniques allow for the efficient extraction of information from states stored in a cavity, illustrating the viability of measuring redundantly encoded states in multi-level systems^13^,^14^. Furthermore, this implementation provides a resource for quantum state tomography and quantum process tomography of continuous variable systems and creates a platform for measurement-based quantum computation and quantum error correction using superconducting cavity resonators^15^. Finally, these features can extend to multi-cavity systems^27^, which will require entanglement detection between continuous variable degrees of freedom and entanglement distribution of complex oscillator states.

## Methods

### Measurement set-up

Experiments are performed in a cryogen-free dilution refrigerator at a base temperature of ∼10 mK. Our output signal amplification chain consists of two stages. A Josephson bifurcation amplifier^31^ operating in a double-pumping configuration^32^,^33^ serves as the first stage, which is followed by a high electron mobility transistor amplifier.

**Figure 5:**
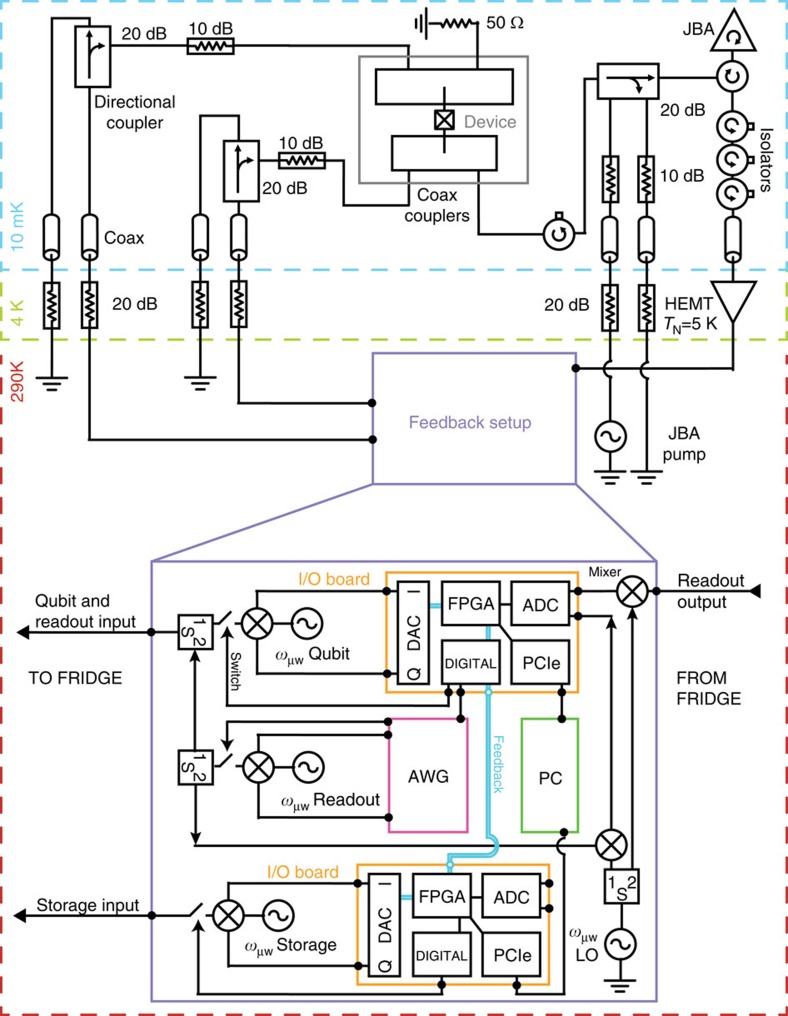
Experiment schematic. Schematic of the experiment shows a two-cavity one-qubit device identical to that used in ref. 22, however with the addition of a feedback set-up for active qubit reset. The feedback set-up uses two input–output (I/O) boards for qubit and storage resonator control and one arbitrary waveform generator (AWG) for readout resonator control. All have a dedicated microwave generator and mixer for amplitude and phase modulation. Each I/O board has five main components: (1) a digital-to-analogue converter (DAC) for pulse generation; (2) digital outputs serving as marker channels; (3) an analogue-to-digital converter (ADC) that samples input signals; (4) an FPGA that demodulates the signals from the ADC and based on predefined thresholds determines the measured qubit state, 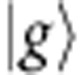 or 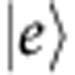 to generate pulses; and (5) a PCIe connection that transfers FPGA data to a computer (PC) for analysis. In this set-up, the top I/O board serves as the master, which accepts the readout signal, returns qubit state information, and using digital output signals, triggers the AWG and the second I/O card given a particular qubit measurement result.

Fabrication techniques of the transmon qubit and the design of storage and readout resonators follow the methods described in ref. 29. The refrigerator wiring (Fig. 5), including the filters and attenuators used, are similar to that of ref. 22, but with the addition of a feedback system, the details of which are discussed in a following section.

### Qubit–cavity parameters

The two-cavity, single-qubit system is well described by the approximate dispersive Hamiltonian:


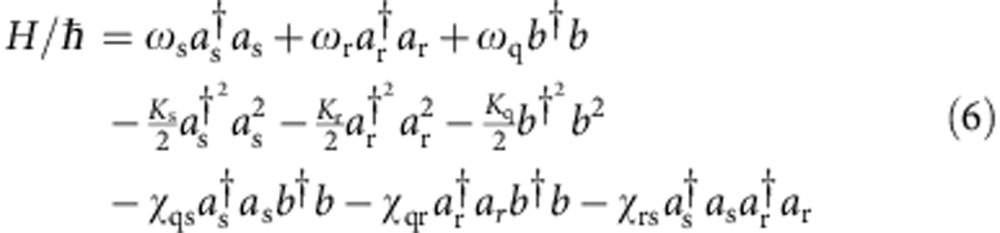


Where *ω*_s_, *ω*_r_ and *ω*_q_ are the storage, readout and qubit transition frequencies, *a*_s_, *a*_r_ and *b* are the associated ladder operators, and *K* and *χ* are the modal anharmonicities and dispersive shifts, respectively. Supplementary Table 1 details the Hamiltonian parameters of our system. The resonant frequency of the readout resonator *ω*_r_/2*π* is determined by transmission spectroscopy. The qubit frequency *ω*_s_/2*π* and storage cavity frequencies *ω*_q_/2*π* are found using two-tone spectroscopy.

Qubit anharmonicity *K*_q_ is measured using two-tone spectroscopy to observe the 0–2 two-photon transition^17^. Storage cavity anharmonicity *K*_s_ is determined by displacing the cavity with a coherent state and observing its time evolution with Wigner tomography. The resulting dynamics are characterized by state reconstruction and *K*_s_ is observed by the state's quadratic dependence of phase on photon number. Finally, we predict the readout cavity anharmonicity *K*_r_ using its approximate dependence on the measured values of *K*_q_ and the qubit-readout dispersive shift *χ*_qr_ (ref. 40).

The dispersive shift between the qubit and the readout resonator *χ*_qr_ is found by taking the difference in frequency between the readout resonance when the qubit is in the ground and excited state. The dispersive shift between the qubit and the storage resonator *χ*_qs_ is found using two methods: photon number-dependent qubit spectroscopy^41^, and observing qubit state revival using Ramsey interferometry^29^. Finally, *χ*_rs_ is predicted using its approximate relationship between *K*_s_ and *K*_r_ (ref. 40).

### Lifetimes and thermal populations

The lifetime of the storage cavity is determined by displacing to a coherent state, waiting a variable length of time, and then applying a qubit rotation conditioned on zero photons in the storage cavity. This allows a measurement of the time-dependent overlap of the cavity state with its ground state 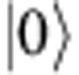 dependent on time. The lifetime of the readout cavity is found from its line width. The thermal population of the qubit is determined from a histogram of one million single-shot measurements of the qubit thermal state, where the signal-to-noise ratio provided by the Josephson bifurcation amplifier allows discrimination between 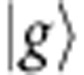 and all states not 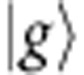. The thermal population of the storage cavity is found by taking the difference between parity measurements of the thermal and vacuum states of the cavity. A vacuum state is prepared by first performing two parity measurements on the thermal state and then post-selecting such that all results give even parity, projecting the thermal state onto 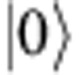. Finally, the known thermal population of the readout cavity is bounded by the dephasing rate Γ_*φ*_ of the qubit: 
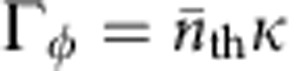
, where 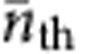 is the readout cavity's thermal occupation and *κ* is the readout single-photon decay rate^42^. Coherence properties are summarized in Supplementary Table 2.

### Measurement fidelities

We define single-shot measurement fidelity as 
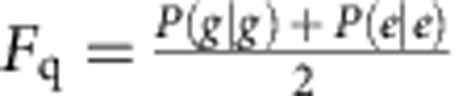
, where *P*(*g*|*g*) and *P*(*e*|*e*) are the probabilities to get 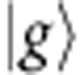 
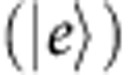
, knowing that we start with 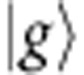 
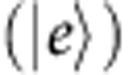
. The state 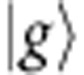 is prepared through purification of the qubit thermal state with real-time feedback (see the following section). Given a preparation of 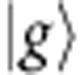, we have a 98.5% chance of measuring 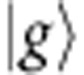 again (*P*(*g*|*g*)=0.985). Likewise, we find *P*(*e*|*e*)=0.975 by preparing 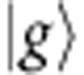 and rotating the state to 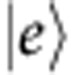. This gives a single-shot measurement fidelity of *F*_q_=98%. We find our cavity parity measurement fidelity by purifying the storage cavity thermal state into 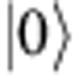 then performing one of two kinds of parity measurement (Supplementary Figs 7 and 8; Supplementary Table 3). We report a parity measurement fidelity for *n*=0 photons as 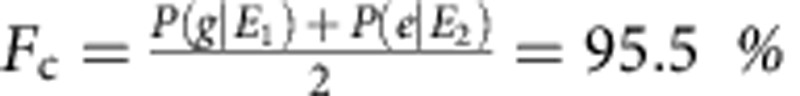, where *P*(*g*|*E*_1_) and (*P*(*e*|*E*_2_)) are the probabilities to measure 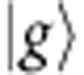 
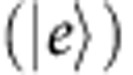
, given that the parity is even for each of the two measurement settings. We expect *F*_c_ to decrease with increasing numbers of photons in the cavity due to single-photon loss during the measurement sequence.

Directly from these readout fidelities, the estimated visibility^43^ for correlated observables 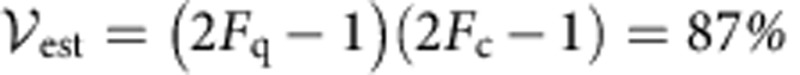. This allows us to predict the maximum Bell violation possible given only measurement inefficiencies 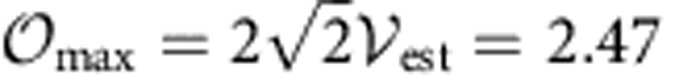. In practice, 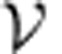 is directly related to the contrast of the joint Wigner function (Supplementary Note 5), which we measure to be 85%. This discrepancy is due to qubit decoherence, which is studied further in Supplementary Note 2, and puts a more conservative estimate for the maximum Bell violation achievable: 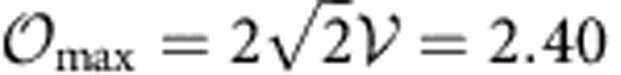.

### I/O control parameters

As shown in Fig. 5, we employ a field-programmable gate array (FPGA) to implement an active feedback scheme. We use an X6-1000M board from Innovative Integration that contains two 1 GS/s analogue-to-digital converters, two 1 GS/s digital-to-analogue converter channels and digital inputs/outputs all controlled by a Xilinx VIRTEX-6 FPGA loaded with custom logic. We synchronize two such boards in a master/slave configuration to have IQ control of both the qubit/storage cavity. IQ control over the readout cavity is performed with a Tektronix AWG, which is triggered by the master board. The readout and reference signals are routed to the analogue-to-digital converters on the master board, whereafter the FPGA demodulates the signal and decides whether the qubit is in 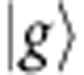 or 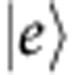. The feedback latency of the FPGA logic (last in, first out LIFO) is 320 ns. Additional delay for active feedback includes cable delay (∼100 ns) and readout pulse length with resonator decay time (320 ns). Thus, in total, the qubit waits *τ*_wait_ ∼740 ns between the time at which photons first enter the readout resonator and the time at which the feedback pulse resets the qubit.

### Implementations of feedback

**Figure 6:**
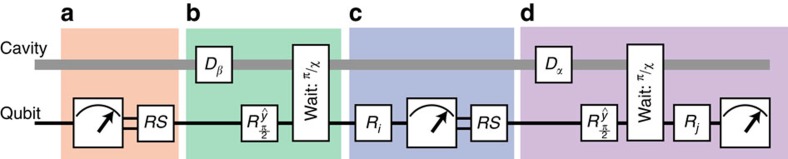
Full quantum circuit diagram. Each experiment is split into four components. (**a**) First, the system is initialized. The qubit state is measured and a qubit pulse 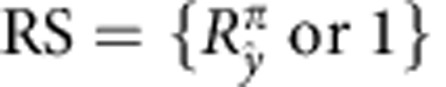 is applied to reset the qubit to 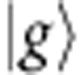. (**b**) Second, the entangled state is created with a cavity displacement *D*_*β*_ and a qubit rotation 
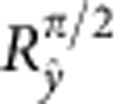
 followed by a 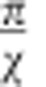 waiting time to produce the entangled state 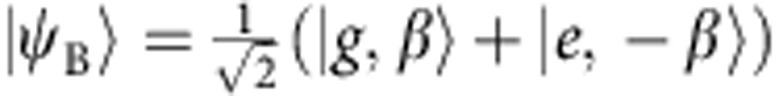. (**c**) Following preparation, a qubit state detection is performed with a pre-rotation *R*_*i*_ (Supplementary Table 3), a measurement, and a qubit reset RS. Finally, we perform a cavity state measurement using Ramsey interferometry, where 
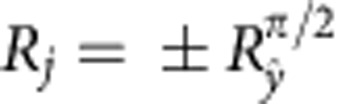
 combined with an initial pre-displacement *D*_*α*_. This maps *P*_*α*_ to the qubit state, which is readout with a subsequent qubit measurement. Correlations are reported as the product of detection events between measurements in **c** and **d**.

Feedback is used three times during a single iteration of the experiment. Before the state preparation (Fig. 6), we purify the qubit state to 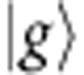 by measuring the qubit and applying a rotation 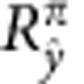 if measured in 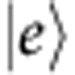. We succeed in preparing 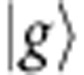 with a probability of 99%. Second, when performing qubit tomography we reset the qubit to 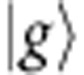 if it is measured to be in 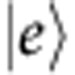. Since we must wait *τ*_wait_ before feedback can be applied, the cavity state will acquire an additional phase *χ*_qs_*τ*_wait_ if the qubit is in 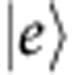. In this case, in addition to resetting the qubit, the FPGA applies an equivalent phase shift on the subsequent Wigner tomography pulse. This feedback implementation does not close the ‘locality' loophole for a CHSH Bell test and therefore cannot be used to test local realism.

### Quantum measurement back action

The sequential measurement protocol allows us to observe the result of quantum measurement back action of the qubit on the cavity state. We prepare the system in a Bell-cat state as in equation (2) and measure along one of the three qubit axes *M*_q_∈{*X*, *Y*, *Z*}. For each measurement, we observe one of two possible outcomes of the projected cavity state 
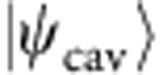
:


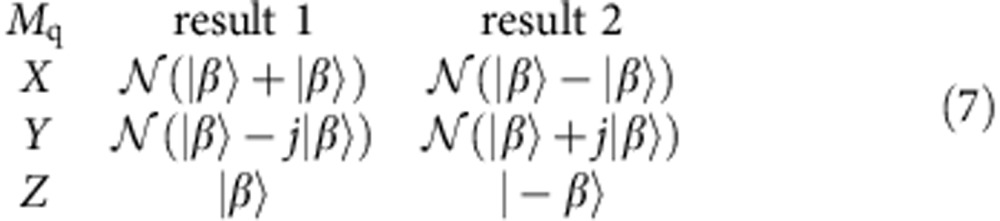


**Figure 7:**
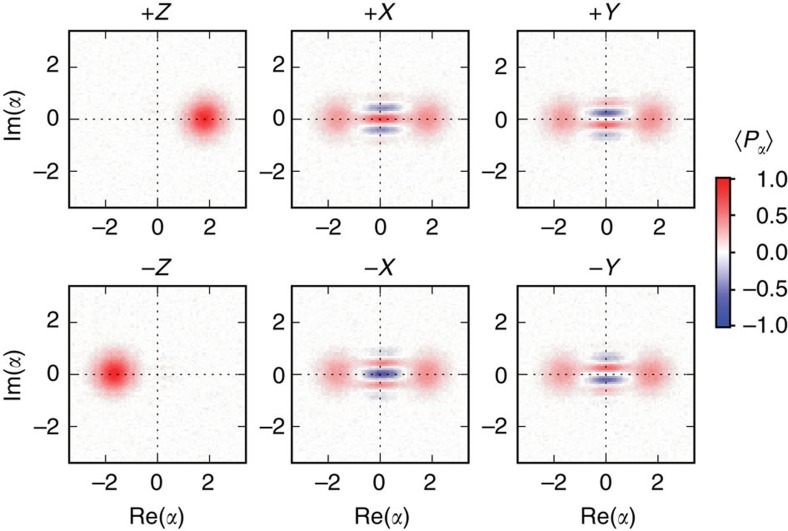
Qubit measurement back action of a Bell-cat state. Shown are the resulting projections of the cavity state when preparing 
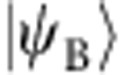
 and measuring the qubit along one of its three axes *Mq*∈{*X*, *Y*, *Z*}. While a measurement along *Z* results in a projected coherent state with opposite phases 
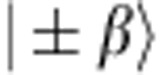
, measuring along the *X* and *Y* axes results in a projected cat state each with different inference fringe phases. Combining these measurements with the probability to obtain each result allows us to construct the state of the entire system and is used to create the joint Wigner function representation in Fig. 2.

See Fig. 7 for each of these projective measurements on the Bell-cat state 
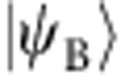
. The method of using strong projective measurements for the create of cat states has been demonstrated in previous works^19^. A second example of quantum measurement back action using an entangled Fock state can be found in Supplementary Fig. 9.

## Additional information

**How to cite this article:** Vlastakis, B. *et al.* Characterizing entanglement of an artificial atom and a cavity cat state with Bell's inequality. *Nat. Commun.* 6:8970 doi: 10.1038/ncomms9970 (2015).

## Supplementary Material

Supplementary Information Supplementary Figures 1-9, Supplementary Tables 1-3, Supplementary Notes 1-15 and Supplementary References
