# Supplementary material for: Characterizing entanglement of an artificial atom and a cavity cat state with Bell's inequality
Source: Nat Commun. 2015 Nov 27;6:8970. doi: 10.1038/ncomms9970 (PMC4674825; doi:10.1038/ncomms9970)
Supplement: Supplementary Information — Supplementary Figures 1-9, Supplementary Tables 1-3, Supplementary Notes 1-15 and Supplementary References [file ncomms9970-s1.pdf]

Supplementary Figures

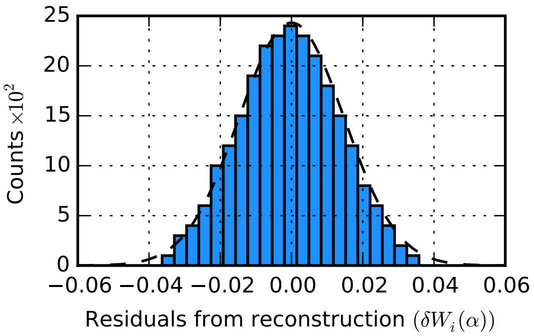

Supplementary Fig. 1: **Histogram of reconstruction residuals.** Plotted are the residuals corresponding to the density matrix reconstruction of the Bell cat state shown in Fig. 2 of the main text. This Histogram shows the distribution of the $25,000$ residuals from the joint Wigner function which gives a Gaussian distribution (mean value $\mu = 7.0 \times 10^{-4}$, standard deviation $\sigma = 0.015$), which agree with our expectation for statistical error $\sigma_{\text{est}} = \frac{1}{\sqrt{N}} \approx 0.015$.

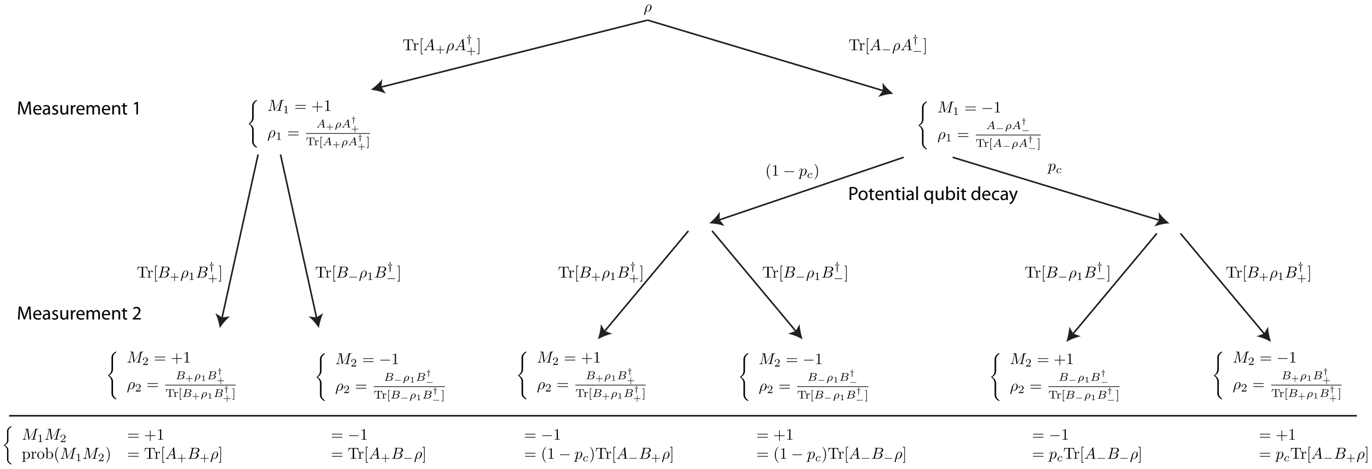

Supplementary Fig. 2: **Measurement trajectories given qubit decoherence.** We can model the behaviour of qubit decoherence in a single measurement trajectory. Qubit decay (which occurs with a probability p) can lead to an improper initialisation of the second detection and in turn produces an incorrect measurement result. This form of detector cross-talk can lead to a reduction in visibility and potential systematic offset of the measured qubit-cavity observable: $\langle AB \rangle \to (1 - p_c) \langle AB \rangle - p_c \langle B \rangle$ (see section 2).

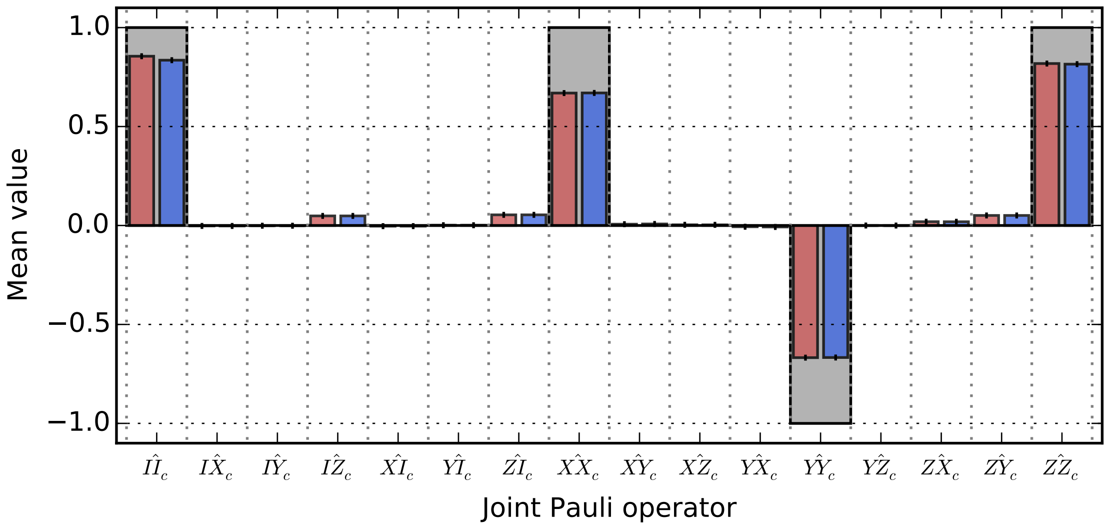

Supplementary Fig. 3: **Reconstructed Pauli sets.** The set of sixteen joint Pauli operators span the two-qubit Hilbert space of the qubit/encoded-qubit state. Shown is the Pauli set for the entangled target state $|\psi_B\rangle$ derived in two ways. (Red) is the reconstructed Pauli set using a density matrix reconstruction of the full quantum state with no normalization constraint, then projecting onto the encoded subspace. (Blue) shows the values discerned from an overlap integral of the measured joint-Wigner functions (Eq. 8). These measurements agree with each other within statistical errors.

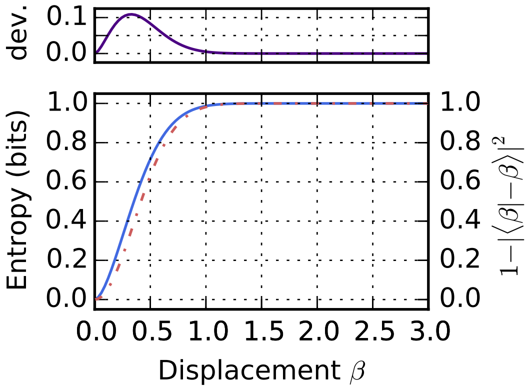

Supplementary Fig. 4: **Informational entropy.** The capacity to store information into a cat state is determined by the orthoganality of its logical states $|\beta\rangle, |-\beta\rangle$. Shown is a comparison between the coherent state overlap (dashed line) and the maximum Von Neumann entropy Eq. 13 (solid line) for this logical encoding. Notice that entropy rapidly approaches one bit for $\beta > 1$, ensuring that information can be reliably encoded into the coherent states with manageable separations.

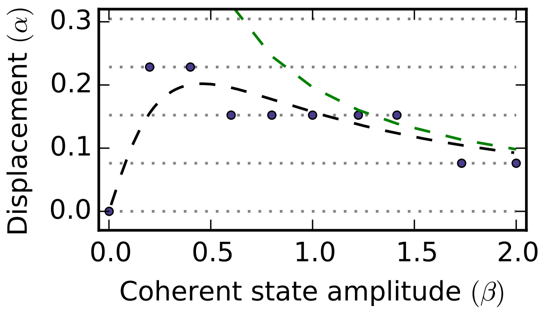

Supplementary Fig. 5: **Optimal displacement for Bell violation.** For performing Bell test #2, the optimal observables to measure maximum correlations depend on the size of the Bell-cat state Eq. 32. The dashed black line shows numerically calculated optimal displacement points as a function of coherent state amplitude β. Shown in circles are the experimentally determined optimal displacement values used to measure a maximum Bell violation. Differences between chosen and ideal values are a result of the discretization of our measurement settings. The dashed green line is the approximate trend $|\alpha_{\mathrm{opt}}| = |\frac{j\pi}{16\beta}|$ for large cat states, which diverge at small β.

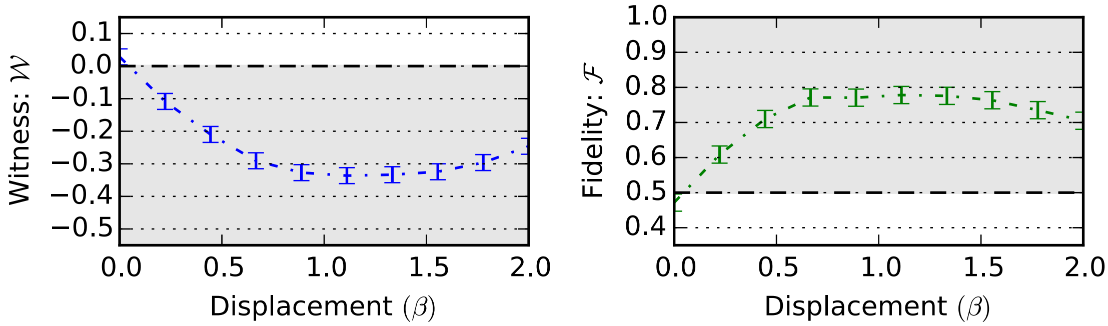

Supplementary Fig. 6: **Entanglement witnesses with cat states.** An entanglement witness and direct fidelity estimation (DFE) are determined by measuring four qubit-cavity correlations. (a) The entanglement witness $\mathcal{W} = II - ZZ - XX + YY$ shows entanglement for all negative values (grey shading). (b) DFE to a target Bell state $\mathcal{F} = II + XX - YY + ZZ$ is also shown where entanglement can be confirmed for values above $\mathcal{F} > 0.5$. Notice that these two witnesses have a much looser bound for entanglement than the CHSH Bell test. Is the standard deviation due to random error limited by the total number of samples N taken at each displacement ($N > 4000$).

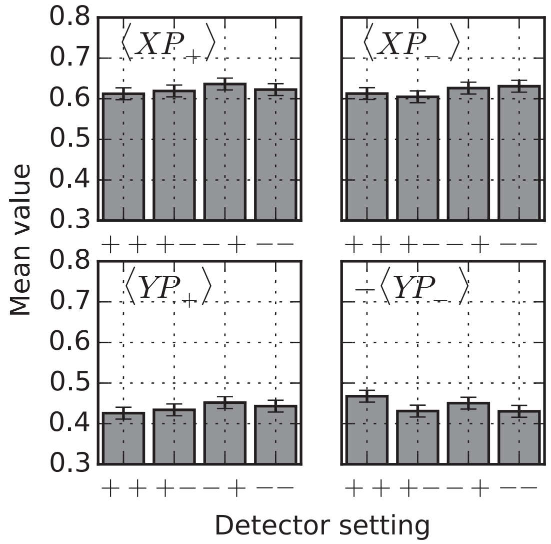

Supplementary Fig. 7: **Observables from each detector setting.** To ensure that a particular detector setting is not producing systematic errors we have not taken into account. We report a Bell test for each detector setting used to observe our maximum violation in test #2. The expectation value of each observable used in that Bell test is shown for the four detector settings used. Significant deviations due to unexpected systematic errors are not observed.

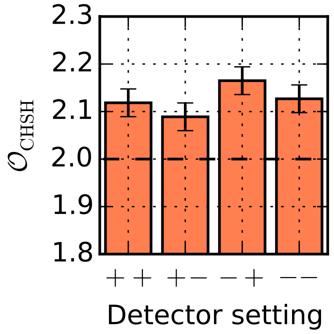

Supplementary Fig. 8: **Bell test for each detector setting.** A Bell test is analyzed for each detector setting to determine the effects of possible systematic errors. Each of these subtests violate Bell's inequality by more than three standard deviations of their statistical error.

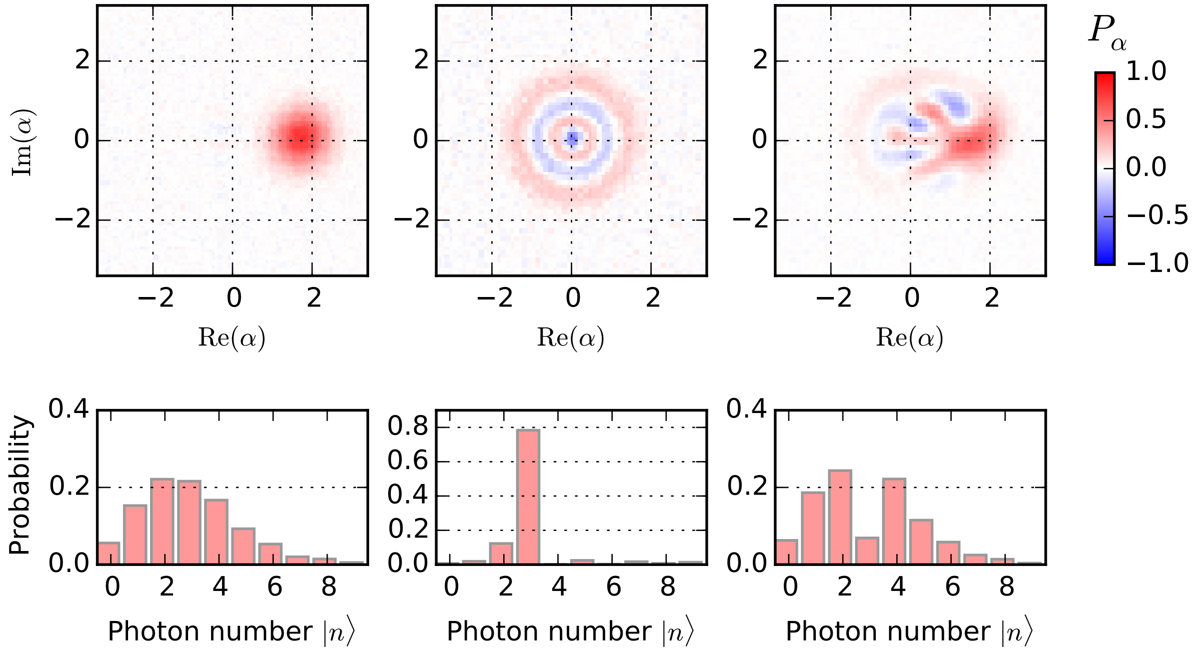

Supplementary Fig. 9: **Qubit measurement back-action for an entangled Fock state.** (a) A measured Wigner function of a coherent state $|\beta\rangle$ where $\beta = \sqrt{3}$ results in a Poissionian photon distribution. Performing a photon-selective qubit rotation on the mth level where $m = 3$ results in an entangled state $|\psi\rangle = C_m |e, m\rangle + \sum_{n \neq m} C_n |g, n\rangle$ where C_n is the coefficient of the nth photon number state $C_n = \langle n|\beta\rangle$. (b) The measured Wigner function of the cavity state after the qubit has been measured in the $-Z$ state results in a 3-photon Fock state. (c) Instead, when a $+Z$ result is obtained the measured cavity state Wigner function is a Fock-state subtracted coherent state $|\psi_c\rangle = \mathcal{N} \sum_{n \neq 3} C_n |n\rangle$.

Supplementary Tables

Term	Measured (Prediction)
$\omega_{\mathrm{q}}/2\pi$	5.7651 GHz
$\omega_{\mathrm{s}}/2\pi$	7.2164 GHz
$\omega_{\mathrm{r}}/2\pi$	8.1740 GHz
$K_{\mathrm{q}}/2\pi$	240 MHz
$K_{\mathrm{s}}/2\pi$	1.5 kHz
$K_{\mathrm{r}}/2\pi$	(2 kHz)
$\chi_{\mathrm{qs}}/2\pi$	1.43 MHz
$\chi_{\mathrm{qr}}/2\pi$	1 MHz
$\chi_{\mathrm{rs}}/2\pi$	(1.7 kHz)

Supplementary Table 1: **Hamiltonian parameters**

	Qubit	Storage	Readout
T_1	10μs	-	-
T_2	10μs	-	-
τ_{cav}	-	55μs	30ns
ground state (%)	90%	$> 98\%$	$> 99.8\%$

Supplementary Table 2: **Coherence and thermal properties**

Qubit		Cavity	
R_i	M_1	R_j	M_2
$\mathbb{1}$	$+Z$	$R_{\hat{y}}^{\pi/2}$	$+P_\alpha$
$R_{\hat{y}}^{\pi}$	$-Z$	$R_{\hat{y}}^{-\pi/2}$	$-P_\alpha$
$R_{\hat{y}}^{\pi/2}$	$+X$		
$R_{\hat{y}}^{-\pi/2}$	$-X$		
$R_{\hat{x}}^{-\pi/2}$	$+Y$		
$R_{\hat{x}}^{\pi/2}$	$-Y$		

Supplementary Table 3: **Table of measurement operators.** As described in Fig. 6 of the main text, pre-rotations before qubit and cavity state measurements determine the measured observable. Shown are the different pre-rotations used and the corresponding measurement operator.

Supplementary Notes

Supplementary Note 1: **Gaussian error statistics.** We perform single-shot measurements that are discriminated into binary results and report measured observables taking the mean of N experimental outcomes. All mean values in this manuscript (including tomography and entanglement witnesses) are reported with $N > 4000$ measurements and the highest measurement fidelity for any joint observable is $\mathcal{F} = (\mathcal{V}+1)/2 = 0.93$ such that $\min[N\mathcal{F}, N(1-\mathcal{F})] > 300 \gg 1$. From this, we can approximate the mean value of all measured observables to follow a gaussian distribution with standard deviation $\frac{1}{\sqrt{N}}$ (Supplementary Fig. 1). In this manuscript, we do not randomly select detector orientations (in order to close a freedom-of-choice loophole) so in turn the number experiments for each detector orientation is pre-determined and equal.

Supplementary Note 2: **Detector cross-talk.** The sequential detection protocol in this experiment uses the same detector to perform first a qubit measurement followed by a cavity measurement. To minimize unwanted systematic errors due to detector cross-talk between measurements, we perform each experiment under four detector setting permutations. Two settings are used for the qubit measurement: a pre-rotation which maps a qubit eigenstate $|\pm\rangle$ to detector values $\pm M_1^q$ and another which maps $|\pm\rangle$ to $\mp M_1^q$. Two settings are used for the cavity measurement: a Ramsey experiment which maps a cavity eigenstate $|\pm\rangle$ to detector values $\pm M_2^c$ and another which maps $|\pm\rangle$ to $\mp M_2^c$ (Supplementary Tab. 3). Each detector setting is performed an equal number of times and results are combined to remove unwanted correlations between detector readings and measured quantum observables. See Sec. 11 for an analysis on the effects of these detector settings on a Bell test.

The dominant form of cross-talk for this experiment is due to qubit state decoherence between measurements. To realize the cavity state measurement, the qubit must be initialized in $|g\rangle$, which we perform using active feedback. Qubit decay can occur during this reset process causing an incorrect initializiation for cavity state detection. We can model this error by observing the possible trajectories of each measurement outcome (Supplementary Fig. 2). This modifies the average measurement of the observable AB where A, B are qubit and cavity operators that can be decomposed into qubit projectors $AB = (A_+ - A_-)B$, where $A_+ + A_- = I$. Due to qubit decay, the measured value $\langle A_+B\rangle$ will be modified to $(1-2p_c)\langle A_+B\rangle$ where p_c is the probability of qubit decay in the time between the first measurement and the feedback rotation. This relation changes the measurement into:

$$\begin{aligned}\langle AB\rangle \to &(1-2p_c)\langle A_+B\rangle - \langle A_-B\rangle \\ =&(1-p_c)\langle A_+B - A_-B\rangle - p_c\langle A_+B + A_-B\rangle \\ =&(1-p_c)\langle AB\rangle - p_c\langle B\rangle\end{aligned} \tag{1}$$

For measuring $B = X_c, Y_c, Z_c$ of the Bell-cat state $|\psi_c\rangle$, we expect $\langle B\rangle = 0$, which gives merely a reduction in the visibility of the observable $\langle AB\rangle$ by a factor$(1-p_c)$ without systematic offsets. We estimate in this experiment that $p_c = 1 - e^{-\frac{\tau_{\text{wait}}}{T_1}} \approx 0.06$. With this justification we can predict the additional loss in visibility $\mathcal{V}$ mentioned in the previous section which gives a visibility $\mathcal{V}_{\text{pred}} = (1-p_c)\mathcal{V} = 82\%$. The experimentally obtained visibility $\mathcal{V}$ is 85%; we believe the discrepency between predicted and measured values is due to an overestimate in the time the qubit is susceptible to energy decay during measurement.

Supplementary Note 3: **Tomography rotation errors.** We observe systematic effects attributed to an amplitude error using the $R_{\tilde{y}}^{\pi}$ operation for pre-rotations used in qubit state tomography. A arbitrary pre-rotation $R^{\theta,\phi} = e^{\frac{i\pi\theta}{2}(\sigma_y\cos\phi + \sigma_x\sin\phi)}$ transforms a qubit measurement along the $\hat{Z}$ axis:

$$\hat{Z} \to \cos\theta\hat{Z} + \sin\theta\cos\phi\hat{X} + \sin\theta\sin\phi\hat{Y} \tag{2}$$

If $\theta \neq 0$ or π, a systematic offset can occur. This is observed in Supplementary Fig. 3 where the contrast of $\langle ZZ_c\rangle$ is reduced and a residual offset in $\langle ZX_c\rangle$ and $\langle ZY_c\rangle$ respectively are produced. From Eq. 2, we predict the following relationship:

$$\langle ZZ_c\rangle \tan\theta = \sqrt{\langle ZX_c\rangle^2 + \langle ZY_c\rangle^2} \tag{3}$$

From measurements, we can approximate the fractional amplitude rotation error $\delta\theta = 4.2\%$. This under-rotation is due to a photon-dependence of the calibrated pulse amplitude. Mitigating this error susceptibility is being explored with composite pulses in future experiments.

Supplementary Note 4: **Calculating observables.** We can represent the density matrix in the excitation number basis:

$$\rho = \sum_{i,j=0}^{1} \sum_{n,m=0}^{N} \rho_{ij}^{nm} |i\rangle \langle j| \otimes |n\rangle \langle m| \tag{4}$$

where ρ_{ij}^{nm} are elements of qubit/cavity density matrix and $|i, j\rangle$ is the qubit state in the excitation basis and $|n, m\rangle$ is the cavity state in the excitation (photon number) basis. From a density operator, one can calculate an observable of the combined system by determining the product of observables from each individual system:

$$\langle AB \rangle = \mathrm{Tr}\left[AB\rho\right] \tag{5}$$

where A, B are operators for the qubit and cavity respectively.

In the joint Wigner function, the qubit basis is the Pauli set $\sigma_i = \{I, \sigma_x, \sigma_y, \sigma_z\}$. For the cavity mode, we choose the displaced photon parity operator $P_\alpha = D_\alpha P D_\alpha^\dagger$ that corresponds to a single point in the cavity state Wigner function. For a truncated Hilbert space (in this experiment $N_{\max} = 12$) and a displacement grid of $\alpha_{\max,\min} = \pm 3.4$ with step size $\Delta\alpha = 0.085$, this measured Wigner function represents an over-complete set of measurements for the cavity mode. The joint Wigner function $W_i(\alpha) = \frac{2}{\pi}\langle \sigma_i P_\alpha \rangle$ is constructed directly from experimental measurements.

A qubit operator A can be written in the Pauli basis $A = \sum_i A_i \sigma_i$ where $A_i = \frac{1}{2}\mathrm{Tr}[A\sigma_i]$ and a bounded cavity observable (see [1] for details) can be represented in continuous-variable basis $B = \frac{1}{\pi}\int B(\alpha) P_\alpha \mathrm{d}^2\alpha$ where $B(\alpha) = \mathrm{Tr}[BP_\alpha]$. Finally, the composite qubit-cavity density matrix can be written as:

$$\rho = \pi \sum_i \int W_i(\alpha) \sigma_i P_\alpha \mathrm{d}^2\alpha \tag{6}$$

Note that for separable states $\rho = \rho_q \otimes \rho_c$, this relation can be split up into their respective discrete and continuous components:

$$\rho = \frac{1}{2}\sum_i \mathrm{Tr}[\rho_q \sigma_i]\sigma_i \otimes 2\pi \int \frac{2}{\pi}\mathrm{Tr}[\rho_c \mathrm{P}_\alpha] P_\alpha \mathrm{d}^2\alpha \tag{7}$$

For any state ρ, we can write the mean value of an observable for the combined system with the following relation:

$$\begin{aligned} \langle AB \rangle &= \mathrm{Tr}\left[AB\rho\right] \\ &= \tfrac{1}{2}\mathrm{Tr}\left[\sum_{i,j}\int A_i B(\alpha) W_j(\alpha')\sigma_i\sigma_j P_\alpha P_{\alpha'} \mathrm{d}^2\alpha \mathrm{d}^2\alpha'\right] \end{aligned} \tag{8}$$

Using the following operator rules $\mathrm{Tr}[\sigma_i\sigma_j] = 2\delta_{ij}$ and $\mathrm{Tr}[P_\alpha P_{\alpha'}] = \delta^2(\alpha - \alpha')$ we can simplify Eq. 8:

$$\langle AB \rangle = \sum_i \int A_i B(\alpha) W_i(\alpha) \mathrm{d}^2\alpha \tag{9}$$

The overlap integral used in this calculation is similar to descriptions of the standard Wigner function [1, 2]. Shown in Supplementary Fig. 3 is a comparison between observables calcuated by Eq. 8 and those determined from a density matrix reconstruction.

Supplementary Note 5: **Detector efficiency.** Under experimental conditions, the measured joint Wigner function is determined with point-by-point measurements of the joint observable $\langle \sigma_i P_\alpha \rangle$. Detector inefficiency results in a reduced visibility $\mathcal{V} \in [0, 1]$ and in turn a reduced contrast of the measured joint Wigner functions $W_i^{\mathrm{meas}}(\alpha) = \mathcal{V} W_i^{\mathrm{ideal}}(\alpha)$. We can determine $\mathcal{V}$ by tracing over both the qubit and cavity states and comparing this to its ideal value $\int W_I^{\mathrm{ideal}}(\alpha)\mathrm{d}^2\alpha = 1$:

$$\mathcal{V} = \int W_I^{\mathrm{meas}}(\alpha)\mathrm{d}^2\alpha \tag{10}$$

where I is the qubit state identity operator. We observe $\mathcal{V} = 85\%$ and attribute this primarily to readout infidelity and qubit decay between the sequential measurements (See Sec. 1).

Supplementary Note 6: **Density matrix reconstruction.** In Fig. 2 of the main text, we show the reconstructed density matrix of a target Bell-cat state $|\psi_{\mathrm{B}}\rangle$. We perform this reconstruction with a-priori assumptions that the cavity state is truncated to twelve occupied photon number states $N_{\max} = 12$, the resulting noise of each averaged measurement is gaussian distributed, and the reconstructed density matrix is postive semidefinite with trace equal to one.

Under these constraints, we perform a least squares regression using a Maximum likelihood estimation [3]. To analyze this regression, we perform residual boostrapping on the reconstructed data set giving bounds on the error statistics of the inferred state.

Supplementary Note 7: **Orthogonality of logical states.** In the main text, we describe encoded qubit states of the cavity where logical states $|0_\mathrm{L}\rangle, |1_\mathrm{L}\rangle$ correspond to coherent states $|\beta\rangle, |-\beta\rangle$. This approximation only holds for coherent states $|\pm\beta\rangle$ that are quasi-orthoganal $|\langle-\beta|\beta\rangle|^2 \ll 1$. To be more precise, we can calculate the maximum Von-Neumann entropy of the encoded space to determine its capacity to store infromation:

$$\begin{aligned} S &= -\mathrm{Tr}\left[\rho_\mathrm{max}\log_2\rho_\mathrm{max}\right] \\ &= -\sum_i \eta_i \log_2 \eta_i \end{aligned} \tag{11}$$

where $\rho_\mathrm{max} = \frac{1}{2}(|\beta\rangle\langle\beta| + |-\beta\rangle\langle-\beta|)$ is the density matrix for a complete mixture of the logical subspace and η is its set of eigenvalues. Rewriting ρ_max in the even/odd cat state basis:

$$\rho_\mathrm{max} = \tfrac{1}{2}(1+e^{-2|\beta|^2})\,|\mathrm{E}\rangle\langle\mathrm{E}| + \tfrac{1}{2}(1-e^{-2|\beta|^2})\,|\mathrm{O}\rangle\langle\mathrm{O}| \tag{12}$$

where $|\mathrm{E}\rangle, |\mathrm{O}\rangle = \frac{1}{\sqrt{2(1\pm e^{-2|\beta|^2})}}(|\beta\rangle \pm |-\beta\rangle)$. Recall that $\langle E|O\rangle = 0$ for all coherent state amplitudes β. This gives the following entropy relation:

$$\begin{aligned} S = &-\tfrac{1}{2}(1+e^{-2|\beta|^2})\log_2\left(\tfrac{1}{2}(1+e^{-2|\beta|^2})\right) \\ &-\tfrac{1}{2}(1-e^{-2|\beta|^2})\log_2\left(\tfrac{1}{2}(1-e^{-2|\beta|^2})\right) \end{aligned} \tag{13}$$

Shown in Supplementary Fig. 4 is the capacity to store information using this encoding scheme. Entropy varies from zero bits to a value asymptotically approaching a single bit with increasing coherent state amplitudes β. The orthoganality betwee logical states $|\langle\beta|-\beta\rangle|^2$ is directly related to this information capacity and serves as a proxy for validating the qubit approximation of the produced cavity state.

Supplementary Note 8: **Encoded state observables.** The coherent state basis chosen in this report to represent the encoded qubit has Pauli operators:

$$\begin{aligned} X_c &= |-\beta\rangle\langle\beta| + |\beta\rangle\langle-\beta| \\ Y_c &= j\,|-\beta\rangle\langle\beta| - j\,|\beta\rangle\langle-\beta| \\ Z_c &= |\beta\rangle\langle\beta| - |-\beta\rangle\langle-\beta| \\ I_c &= |\beta\rangle\langle\beta| + |-\beta\rangle\langle-\beta| \end{aligned} \tag{14}$$

Here, we will show that the operators expressed in Eq. 4 of the main text, approximate these encoded Pauli operators. Assuming $\langle\beta|-\beta\rangle \ll 1$, we have the following photon-number parity P relations:

$$\begin{aligned} \langle\beta|P_0|\beta\rangle &= \langle\beta|-\beta\rangle \ll 1 \\ \langle\beta|P_0|-\beta\rangle &= \langle\beta|\beta\rangle = 1 \\ \langle\beta|P_\alpha|\beta\rangle &= \langle\beta-\alpha|\alpha-\beta\rangle \ll 1 \\ \langle\beta|P_\alpha|-\beta\rangle &= e^{2(\alpha\beta^*-\alpha^*\beta)}\langle\alpha|-\alpha\rangle \end{aligned} \tag{15}$$

where $P_\alpha = D_\alpha P D_{-\alpha}$ for some displacement amplitude α. Now taking the projector $M = |\beta\rangle\langle\beta| + |-\beta\rangle\langle-\beta|$, we derive the encoded state's Pauli Operators from the cavity state observables reported in Eq. 4 of the main text:

$$\begin{aligned} MP_0M^\dagger &\approx |-\beta\rangle\langle\beta| + |\beta\rangle\langle-\beta| \\ MP_\beta M^\dagger &\approx |\beta\rangle\langle\beta| \\ MP_{-\beta}M^\dagger &\approx |-\beta\rangle\langle-\beta| \\ MP_{\frac{j\pi}{8\beta}}M^\dagger &\approx j\,|-\beta\rangle\langle\beta| - j\,|\beta\rangle\langle-\beta| \end{aligned} \tag{16}$$

Putting these relationships together, as in Eq. 4, builds the encoded state observables $\{X_c, Y_c, Z_c, I_c\}$ and reveals that these observables can be efficiently measured using Wigner tomography. I_c and Z_c require a comparison between two different observables. For true single-shot readout of these logical observable Z_c, measuring a single value in the cavity state Husimi-Q distribution $Q(\beta) = \frac{1}{\pi}\langle\beta|\rho|\beta\rangle$ can be employed where $Z_c = 2\pi Q(\beta) - 1$. This is being explored in future experiments.

Supplementary Note 9: **Encoded state Pauli set.** We can represent the two qubit Bell state shown in Fig. 2 of the main text as a list of two-qubit correlations. The complete set constitutes the permutation of each of the single qubit Pauli set $\{I, X, Y, Z\}$. We can determine the two-qubit Pauli set from the complete reconstructed qubit-cavity state and projecting onto the encoded basis of $\{I_c, X_c, Y_c, Z_c\}$. Supplementary Fig. 3 shows the resulting two-qubit Pauli set for the transmon qubit and an encoded qubit in the cavity mode, a variant of the reduced density matrix representation shown in Fig. 2 of the main text.

Supplementary Note 10: **Encoded state preparation.** We can diagnose errors that can occur during state preparation from the reconstructed Pauli set. The dominant nonideal effects we explore are qubit decay during preparation and single-qubit rotation error.

During state preparation, the product state $|\psi\rangle = \frac{1}{\sqrt{2}}(|g\rangle + |e\rangle) \otimes |\beta\rangle$ is initialized. Under the dispersive interaction, the system evolves into the entangled state $\frac{1}{\sqrt{2}}(|g,\beta\rangle + |e,-\beta\rangle)$. To describe the effects of T_1 decay, we can look at the diagonal elements of the reduced density matrix after the entangling evolution of the dispersive interaction:

$$\begin{aligned}\mathrm{diag}[\rho] = \tfrac{1}{2}\{\, &|g,\beta\rangle\langle g,\beta| + e^{-\gamma}|e,-\beta\rangle\langle e,-\beta| \\ &+ \sum_k C_k |g,\alpha_k\rangle\langle g,\alpha_k| \,\}\end{aligned} \tag{17}$$

where $\alpha_i = \beta e^{j\chi t_k}$ represents coherent states when a jump occured at time t_k and $\gamma = \frac{\pi}{\chi T_1}$. Projecting onto the logical basis (here we will aproximate $|\langle\alpha_k|\beta\rangle|^2 \ll 1$) produces the resulting scaling on the joint Pauli measurements for the ideal state:

$$\begin{aligned}\langle II_c\rangle &\propto \tfrac{1}{2}\left(1 + e^{-\gamma}\right) \quad \langle ZI_c\rangle \propto \tfrac{1}{2}\left(1 - e^{-\gamma}\right) \\ \langle ZZ_c\rangle &\propto \tfrac{1}{2}\left(1 + e^{-\gamma}\right) \quad \langle IZ_c\rangle \propto \tfrac{1}{2}\left(1 - e^{-\gamma}\right)\end{aligned} \tag{18}$$

This gives us an approximate method to predict our ability to prepare a state that is within the logical subspace given our experimental parameters, $\langle II_c\rangle = 0.99$. Our measured value taking into account detector inefficiencies produces $\langle II_c\rangle = 0.98$.

The preparation of this entangled system is also sensitive to the amplitude of the initial qubit rotation $Y/2 = R_{\hat{y}}^{\frac{\pi}{2}}$. The angle of rotation θ will determine the prepared state as:

$$|\psi\rangle = \mathcal{N}\{\cos\tfrac{\theta}{2}|g,\beta\rangle + \sin\tfrac{\theta}{2}|e,-\beta\rangle\} \tag{19}$$

For the states prepared nearly as $|\psi_{\mathrm{B}}\rangle$, $\theta \approx \frac{\pi}{2}(1 - \delta\theta)$ and will result in the following modification of the joint Pauli measurements for an ideal state:

$$\begin{aligned}\langle ZI_c\rangle &\propto \tfrac{\pi}{4}\delta\theta \\ \langle IZ_c\rangle &\propto \tfrac{\pi}{4}\delta\theta\end{aligned} \tag{20}$$

From the measurements in Supplementary Fig. 3, we can determine that the relative error for the rotation angle in our prepration rotation to be $\delta\theta \approx 2.8\%$.

Supplementary Note 11: **Bell test analysis.** The main text reports CHSH Bell tests composed of two qubit observables A, B and two cavity observables A_c, B_c, correlated such that:

$$\mathcal{O} = \langle AA_c\rangle + \langle AB_c\rangle - \langle BA_c\rangle + \langle BB_c\rangle \tag{21}$$

We perform two variants of this test on the state $|\psi_{\mathrm{B}}\rangle$.

Test #1 Model: In the first test we choose qubit cavity observables Z_c, X_c and qubit observables $Z(\theta), X(\theta)$ where:

$$Z(\theta) = Z\cos\tfrac{\theta}{2} - X\sin\tfrac{\theta}{2} \qquad\qquad X(\theta) = X\cos\tfrac{\theta}{2} + Z\sin\tfrac{\theta}{2} \tag{22}$$

This angle θ corresponds to a rotation of the qubit state before detection. In Fig. 4a of the main text, we plot $\mathcal{O}$ for each of the four permutations of the joint observables and find a maximum Bell violation for an angle $\theta = -\frac{\pi}{4}$ giving observables:

$$\begin{aligned}A &= \tfrac{X+Z}{\sqrt{2}}; \quad B = \tfrac{X-Z}{\sqrt{2}} \\ A_c &= Z_c; \quad\;\; B_c = X_c\end{aligned} \tag{23}$$

As shown in Fig. 4c of the main text, we can model the effects of photon loss and measurement inefficiency on the maximum violation. For the ideal case, an overlap of the coherent state superposition reduces contrast in $\langle AZ_c\rangle$ and $\langle BZ_c\rangle$ and will limit the maximum Bell signal:

$$\mathcal{O}_{\mathrm{ideal}} = \sqrt{2}(2 - e^{-8|\beta|^2}) \tag{24}$$

Measurement inefficiency will reduce the contrast of this maximum Bell signal which we expect to go as the visibility $\mathcal{V}$:

$$\mathcal{O}_{\text{vis}} = \sqrt{2}\mathcal{V}(2 - e^{-8|\beta|^2}) \tag{25}$$

Photon loss will also have an effect on the maximum Bell signal by reducing the measured contrast of all correlations for $\langle AX_c\rangle$ and $\langle BX_c\rangle$. This produces the an amplitude dependent maximum Bell Signal:

$$\mathcal{O}_{\text{loss}} = \sqrt{2}(1 - e^{-8|\beta|^2} - e^{-2|\beta|^2\gamma}) \tag{26}$$

where $\gamma = \frac{t_{\text{eff}}}{\tau_s}$ such that τ_s is the photon decay time constant and t_{eff} is the effective time to create and measure the Bell-cat state. Finally taking into account both visibility and photon loss produces the expected maximum Bell signal:

$$\mathcal{O}_{\text{pred}} = \sqrt{2}\mathcal{V}(1 - e^{-8|\beta|^2} - e^{-2\gamma|\beta|^2}) \tag{27}$$

This predicted Bell signal is shown in Fig. 4 of the main text using the measured joint-Wigner contrast $\mathcal{V} = 0.85$ and time between cavity state creation and detection $t_{\text{eff}} = 1.24$ μs.

Test #2 Model: In the second test, we choose qubit observables X, Y and cavity observables $X_c(\alpha), Y_c(\alpha)$ where:

$$\begin{aligned} X_c(\alpha) &= D_{j\alpha}P_0D^\dagger_{j\alpha} \approx X_c\cos\tfrac{\alpha}{4\beta} + Y_c\sin\tfrac{\alpha}{4\beta} \\ Y_c(\alpha) &= D_{j\alpha}P_{\frac{j\pi}{8\beta}}D^\dagger_{j\alpha} \approx Y_c\cos\tfrac{\alpha}{4\beta} - X_c\sin\tfrac{\alpha}{4\beta} \end{aligned} \tag{28}$$

Where the displacement amplitude α corresponds to an approximate rotation of the encoded cavity state before detection. In Fig. 4b, we plot $\mathcal{O}$ for each of the four permutations of the joint observables and find a maximum Bell violation for a displacement $\alpha = 0.15$ for $\beta = 1$ which produces the approximate observables:

$$\begin{aligned} A &= X; & B &= Y \\ A_c &= \tfrac{X_c+Y_c}{\sqrt{2}} & B_c &= \tfrac{X_c-Y_c}{\sqrt{2}} \end{aligned} \tag{29}$$

Shown in Fig. 4c of the main text, we can also model the effects of photon loss and measurement inefficiency for the second test. The ideal case is the result of four summed joint Wigner values represented as:

$$\mathcal{O}_{\text{ideal}} = 2(\cos 4\alpha_0\beta + \sin 4\alpha_0\beta)e^{-2|\alpha_0|^2} \tag{30}$$

where α_0 is an optimal displacement for maximum violation which can be calculated from Eq. 32 and in detail in Ref. [4]. Taking into account photon loss and measurement inefficiency produces the following relationship:

$$\mathcal{O}_{\text{pred}} = 2\mathcal{V}e^{-2\gamma|\beta|^2}(\cos 4\alpha_0\beta + \sin 4\alpha_0\beta)e^{-2|\alpha_0|^2} \tag{31}$$

This predicted Bell signal is shown in Fig. 4b of the main text using the measured joint-Wigner contrast $\mathcal{V} = 0.85$ and an effective time $t_{\text{eff}} = 1.24$ μs.

Supplementary Note 12: **Optimal measurements for encoded observables.** Eq. 3 of the main text describes the ideal observables to efficiently determine an encoded qubit state observable using a superposition state with $|\beta| \gg 1$. In fact, the optimal measurement for particular observables will be further modified for smaller coherent displacements.

For the second CHSH experiment, the optimal observable $P_{\pm j\alpha_0} \sim \frac{1}{\sqrt{2}}(\hat{X}_c \pm \hat{Y}_c)$ follows the relation:

$$\frac{\beta - \alpha_0}{\beta + \alpha_0} = \tan 4\alpha_0\beta \tag{32}$$

where α_0 is the amplitude for a coherent displacement $D_{j\alpha_0}$ to perform the measurement $P_{j\alpha_0}$ given β. Further details are discussed in Ref. [4]. In the large β limit, the observable corresponds to the encoded qubit state observable $\frac{1}{\sqrt{2}}(\hat{X}_c + \hat{Y}_c)$ and follows the relationship $P_{\alpha=\frac{j\pi}{16\beta}}$ as related in Eq. 4 of the main text. Shown in Supplementary Fig. 5 is the predicted and chosen optimal values for a maximum CHSH Bell signal.

Supplementary Note 13: **Two-qubit entanglement witnesses.** Two qubit entanglement can also be quantified by an entanglement witness $\mathcal{W} = II_c - XX_c + YY_c - ZZ_c$ [5] for a Bell state $|\psi\rangle = \frac{1}{\sqrt{2}}(|gg\rangle + |ee\rangle)$. The witness 'confirms' entanglement for all observations of $\langle\mathcal{W}\rangle < 0$. Shown in Supplementary Fig. 6, we report $\mathcal{W}$ (as well as its corresponding direct fidelity estimation $\mathcal{F}$) as a function of coherent state amplitude β using the optimal displacements described in Supplementary Fig. 5. As expected, entanglement is not detected for a $\beta = 0$ coherent state (a product state $\frac{1}{\sqrt{2}}(|g\rangle + |e\rangle) \otimes |0\rangle$).

Supplementary Note 14: **Bell test for each detector setting.** We analyze the systematic errors that can occur from a particular detector setting. Shown in Supplementary Fig. 7 are the observables used to calculated a Bell violation using test #2 for each of the four detector settings Sec. 1. Systematic errors are shown to be within statistical bounds of the experiment and each detector setting violates Bell's inequality by at least three standard deviations; see Supplementary Figs. 7 and 8. In the main text, we report measurements from the combined data set resulting in smaller statistical error and a stronger violation of Bell's inequality.

Supplementary Note 15: **Sequential detection with Fock states.** The sequential measurement protocol allows us to observe quantum measurement back-action of the qubit on the cavity state. This is not restricted to coherent state superpositions as shown in the main text. For example, we can prepare the system in a state such that the qubit state $|e\rangle$ is correlated with the mth photon Fock state $|m\rangle$ of a coherent state $|\beta\rangle$ (in this example $m = 3$ photons and $\beta = \sqrt{3}$). This can be written as:

$$|\psi\rangle = C_m |e, m\rangle + \sum_{n\neq m} C_n |g, n\rangle \tag{33}$$

where $C_m = \langle m|\beta\rangle$. Shown in Supplementary Fig. 9, when the qubit is measured along the $\hat{Z}$ axis we observe a change in photon statistics such that a $+1$ event projects the cavity onto the state $|\psi_{\text{cav}}\rangle = \mathcal{N}(|\beta\rangle - C_m |m\rangle)$ and a -1 event projects onto the Fock state $|\psi_{\text{cav}}\rangle = |m\rangle$.

Supplementary References

[1] Cahill, K. E. & Glauber, R. J. Density operators and quasiprobability distributions. *Physical Review* **177**, 1882 (1969).

[2] Haroche, S. & Raimond, J.-M. *Exploring the Quantum: Atoms, Cavities, and Photons* (Oxford University Press, 2006).

[3] Smolin, J. A., Gambetta, J. M. & Smith, G. Efficient method for computing the maximum-likelihood quantum state from measurements with additive Gaussian noise. *Physical Review Letters* **108**, 070502 (2012).

[4] Park, J., Saunders, M., Shin, Y.-i., An, K. & Jeong, H. Bell-inequality tests with entanglement between an atom and a coherent state in a cavity. *Physical Review A* **85**, 022120 (2012).

[5] Horodecki, R., Horodecki, P., Horodecki, M. & Horodecki, K. Quantum entanglement. *Reviews of Modern Physics* **81**, 865–942 (2009).
